# Quality over quantity: early performance predicts suturing proficiency in robotic surgery simulation

**DOI:** 10.1007/s11701-026-03702-0

**Published:** 2026-07-21

**Authors:** Júlia Iaroseski, André Vicente Bigolin, Sofia Galvão Lima, Silvio César Cazella

**Affiliations:** 1 Universidade Federal de Ciências da Saúde de Porto Alegre, Porto Alegre, Brazil; 2 Irmandade da Santa Casa de Misericórdia de Porto Alegre, Porto Alegre, Brazil

**Keywords:** Robotic surgery, Simulation training, Surgical education, Pedagogical trajectory, Suturing, Proficiency

## Abstract

Robotic surgery training increasingly relies on simulation-based curricula and objective performance metrics; however, it remains unclear whether early simulator performance predicts later proficiency in complex robotic tasks. This retrospective observational cohort study analyzed simulation training data from the Simulation Center of Santa Casa de Porto Alegre, Brazil, collected between 2020 and 2024. Early-stage training metrics included time, repetitions, and mean performance scores in the Basic, Essential, and Fundamentals modules, while the primary outcome was the final simulator-derived Suture Score. Correlation analyses were performed to assess the association between prior training trajectory and final suturing performance. Fifty-seven participants were included. Mean performance scores in the Basic and Fundamentals modules were significantly associated with shorter suturing time (r = −0.269, p = 0.043 and r = −0.325, p = 0.014, respectively). Mean score in the Basic module was also associated with a higher final Suture Score (r = 0.277, p = 0.037). In contrast, total simulation time and number of repetitions showed no significant correlation with final proficiency (all p > 0.05). These findings suggest that early simulator performance scores may be more informative than training duration alone in predicting proficiency in complex robotic suturing and support the development of individualized, performance-based robotic training pathways.

**Supplementary Information:**

The online version contains supplementary material available at 10.1007/s11701-026-03702-0.

## Introduction

Robotic surgery has become an increasingly important component of minimally invasive surgery, offering technical advantages such as three-dimensional visualization, improved ergonomics, greater dexterity, and enhanced precision when compared with conventional laparoscopy [1, 2]. As robotic platforms have expanded across specialties, training requirements have become more complex, particularly because robotic surgery demands psychomotor and cognitive skills that are not fully transferable from open or laparoscopic surgery alone [3, 4].

Traditionally, technical skills were acquired in the operating room under an apprenticeship model summarized as “see one, do one, teach one.” Work-hour restrictions, patient-safety imperatives, and ethical concerns about learning on patients have progressively shifted technical skill acquisition toward the preclinical setting [5]. Within this shift, simulation-based training has become a central strategy in robotic surgical education, with evidence of skill acquisition and partial transfer to operative performance [6, 7]. Virtual reality (VR) simulators provide a controlled, risk-free environment for repeated practice with objective feedback [6, 8], and meta-analytic evidence shows that VR training improves objective performance scores and reduces task-completion times compared with no additional training, performing comparably to dry-lab training [9]. These platforms generate detailed metrics — including time, economy of motion, and collisions — enabling standardized, measurable assessment of technical development [7, 10].

The educational value of these metrics depends on the validity of the underlying platform. Several VR simulators used in robotic training, including the RobotiX Mentor, have accumulated face, content, and construct validity evidence for both basic-skill and procedural modules [11–13]. Interpretation of simulator performance is further informed by established educational frameworks. Cognitive load theory holds that learning depends on managing the limited capacity of working memory, so that instructional designs which optimize load facilitate schema formation and skill automation [14, 15]. Complementarily, the theory of deliberate practice proposes that expert performance arises not from accumulated exposure alone, but from structured, goal-directed practice with feedback [16, 17].

These frameworks underpin the evolution of curricular models. Traditional approaches often relied on fixed training time or procedural volume, whereas proficiency-based progression requires trainees to advance only after reaching predefined performance benchmarks [18, 19]. Proficiency-based and adaptive models have shown improved efficiency and better technical outcomes when compared with less structured or self-directed approaches [19–21]. However, these models remain heterogeneous in practice, and there is no clear consensus regarding the ideal sequence or educational value of individual simulator tasks [22].

A central unresolved question is whether greater dedication to basic simulation tasks translates into superior performance in more complex robotic tasks. Basic exercises such as clutching, camera control, peg transfer, and elementary dissection are widely incorporated because they are presumed to establish foundational psychomotor skills [7, 22], whereas complex tasks such as suturing require higher-order integration of depth perception, bimanual coordination, force control, and precision [23]. Performance in specific technical substeps can influence subsequent execution within complex tasks [23], and objective performance metrics may help identify patterns of progression [8, 24]. Importantly, evidence from other domains suggests that cumulative exposure alone is a poor proxy for competence: the number of procedures performed does not reliably predict arthroscopic proficiency [25], and baseline simulator performance appears more closely related to psychomotor and visuospatial aptitude than to sheer volume [26, 27]. Nevertheless, the real-world relationship between time spent, repetition, or mean performance in basic modules and subsequent proficiency in complex tasks remains insufficiently understood.

Many robotic training centers continue to organize simulation exposure predominantly according to workload or fixed hours rather than individualized, performance-guided trajectories, and accelerated, metrics-driven protocols have been proposed to optimize this process [28]. While simulation is widely recognized as an essential educational tool, there is a paucity of studies investigating which specific early performance metrics best predict success in advanced surgical tasks. Understanding this relationship is fundamental for optimizing proficiency-based training pathways. Therefore, this study aimed to determine if performance metrics in early simulation modules predict proficiency in advanced robotic suturing.

### Study design and setting

This retrospective observational cohort study was conducted using real-world data previously generated during robotic surgery simulation training at the Simulation Center of Santa Casa de Porto Alegre, Brazil, between January 2020 and December 2024. The study investigated whether metrics derived from the early stages of simulator training were associated with subsequent performance in a complex suturing task, and was reported in accordance with the STROBE statement.

### Simulator and psychometric properties

All training modules were performed on the RobotiX Mentor virtual reality simulator (Simbionix/Surgical Science, Gothenburg, Sweden). This platform has accumulated validity evidence in robotic surgery training: face, content, and construct validity have been demonstrated for both basic-skill and full-procedure modules [11–13]. Predictive validity is supported by significant correlations between simulator-derived scores and prior robotic operative experience [12]. Reliability of the platform’s performance metrics has also been reported, with test–retest reliability ranging from 0.75 to 0.90 across one to three test repetitions [12]. Because all performance metrics analyzed in the present study were automatically generated by this single validated platform according to its predefined benchmarks, metric definitions and scoring were standardized across all participants.

### Participants

The study population comprised licensed physicians and surgeons undergoing formal robotic simulation training at the Simulation Center of Santa Casa de Porto Alegre; undergraduate medical students were not eligible. Participants were identified from all users recorded in the institution’s MentorLearn database for the RobotiX Mentor simulator during the study period.

Eligibility was defined by the following inclusion criteria: (i) complete digital training records in the MentorLearn (RobotiX Mentor) system; (ii) at least three fully recorded sessions of basic tasks with individualized task registration; (iii) at least one completed complex task with full technical recording; and (iv) a minimum interval of 7 days between the first and last recorded session. The 7-day minimum interval and the three-session threshold were established to ensure a meaningful longitudinal training exposure and to exclude users with isolated, incidental, or single-use access to the platform, so that the analytic cohort represented participants undergoing a formal training process rather than sporadic simulator use.

Participants were excluded if they met any of the following criteria: (i) no completed complex task with full technical data for outcome assessment; (ii) incomplete sessions or major recording failures; (iii) fewer than three recorded sessions; (iv) duplicated or conflicting identifiers; or (v) part of the training performed on a different simulator platform, in order to preserve consistency of the training environment and performance metrics.

Of 362 user records initially screened, 89 met the inclusion criteria. Three users were excluded because part of their training had been performed on another simulator platform, and additional records were excluded for duplicated or conflicting identifiers, incomplete or unreliable recordings, or absence of a complex task with complete technical data. The final analytic sample comprised 57 participants.

### Data sources and study variables

Training data were extracted from MentorLearn (RobotiX Mentor) and organized into structured datasets in which each row represented one simulation session and each column represented one technical variable generated by the simulator. A detailed description of the dataset structure, including variable names, data types, and definitions, is provided in the Supplementary Appendix.

The pedagogical trajectory was characterized using objective simulator-derived variables from the native training pathway of the platform. The classification into Basic, Essential, and Fundamentals modules was not created by the investigators but followed the original structure of the simulator curriculum. The analyzed variables included time spent in each module, number of repetitions, total simulation time, proportional time spent in each module, and mean performance scores when available. Score availability was platform-defined, which is why mean scores were available for the Basic and Fundamentals stages but not for the Essential stage in the analytical dataset. Only the native Basic, Essential, and Fundamentals modules were considered in the trajectory analysis. Time spent on more complex tasks, such as anastomosis and dissection exercises, was excluded because performance in these activities may be influenced by specialty-specific skills and could therefore introduce bias.

The primary outcome was performance in the final complex suturing task, represented by the Suture Score, a composite score automatically generated by the native simulator platform according to its own predefined benchmarks. In this study, the Suture Score was derived from the following component metrics: total suturing time, percentage of accurate needle passages, number of unnecessary needle piercing points, knot tail length deviation, time the needle was held outside the visible field, excessive force leading to suture breakage, and total number of knots. These component variables were also analyzed individually as secondary technical outcomes.

### Sample size

Although this was a retrospective study, the original protocol included a statistical power calculation based on the main study hypothesis, namely the association between time dedicated to basic tasks and performance in complex tasks. Assuming an expected moderate correlation of *r* = 0.30, a significance level of 5%, and a statistical power of 80%, the estimated minimum sample size was 85 participants, as calculated using G*Power 3.1. Because the eligible dataset contained fewer individuals than this target, all available cases meeting the predefined selection criteria were included in the final analysis. This constraint is acknowledged as a limitation with respect to the statistical power to detect smaller effects.

### Statistical analysis

Continuous variables were summarized as mean and standard deviation. Normality was assessed using the Kolmogorov–Smirnov test. Participants were stratified according to final Suture Score into a lower-performance group (scores 1–5) and a higher-performance group (scores 6–7). Comparisons between groups were performed using the Mann–Whitney test. Correlations between pedagogical trajectory variables and suturing outcomes were assessed using Spearman’s correlation coefficient. The significance level was set at 0.05, and all analyses were performed using IBM SPSS Statistics for Windows, Version 25.0 (IBM Corp., Armonk, NY, USA).

### Ethics

This study was performed in line with the principles of the Declaration of Helsinki. Approval was granted by the Ethics Committee of Santa Casa de Porto Alegre, Brazil (CAAE 89831725.0.0000.5335).

## Results

A total of 57 participants met the inclusion criteria and were analyzed. This section first summarizes the participants’ training trajectory and their performance in the final suturing task (Table 1), then compares training-trajectory variables between lower- and higher-performing groups (Table 1), and finally examines the correlations between early training metrics and final suturing outcomes (Table 2). Overall, the analyses revealed a consistent pattern: quantitative exposure variables, such as total simulation time and number of repetitions, were not significantly associated with final performance, whereas mean performance scores obtained during the early training stages showed the most relevant associations.

**Table 1:** Training trajectory variables and final suturing performance according to Suture Score group

Variable	Overall (*n* = 57)	Suture Score 1–5 (*n* = 39)	Suture Score 6–7 (*n* = 18)	*p* value
**Early training trajectory**				
Time in Basic, min	362.9 ± 207.6	338.5 ± 154.9	415.8 ± 289.7	0.613
Repetitions in Basic	110.1 ± 54.7	105.3 ± 50.4	120.7 ± 63.2	0.425
Mean score in Basic¹	83.6 ± 8.0	83.1 ± 8.6	84.6 ± 6.8	0.328
Time in Essential, min	118.9 ± 207.7	128.5 ± 240.5	98.2 ± 109.7	1.000
Repetitions in Essential	23.1 ± 19.7	21.6 ± 15.9	26.4 ± 26.4	0.777
Time in Fundamentals, min	109.4 ± 82.1	111.9 ± 68.3	103.9 ± 108.3	0.144
Repetitions in Fundamentals	31.5 ± 26.7	31.7 ± 20.0	31.2 ± 38.2	0.213
Mean score in Fundamentals¹	66.5 ± 19.6	69.2 ± 13.3	60.8 ± 28.7	0.870
Total simulation time, min	642.0 ± 268.7	670.9 ± 245.8	579.3 ± 310.8	0.140
**Proportional distribution of simulation time**				
Basic time, %	51.0 ± 17.0	49.8 ± 17.8	53.7 ± 15.2	0.450
Essential time, %	13.0 ± 12.5	13.7 ± 14.1	11.4 ± 7.9	0.850
Fundamentals time, %	15.2 ± 9.5	16.5 ± 10.1	12.3 ± 7.8	0.167
**Final suturing task metrics**				
Total suturing time, s	356.6 ± 337.8	420.9 ± 382.8	217.3 ± 134.6	< 0.001
Accurate needle passages, %	81.8 ± 26.5	73.7 ± 28.7	99.4 ± 1.6	< 0.001
Unnecessary needle piercing points	8.9 ± 13.0	11.4 ± 15.0	3.3 ± 2.6	0.001
Knot tail length deviation, mm	28.4 ± 30.6	32.6 ± 35.0	19.1 ± 14.8	0.319
Time with needle outside the visible field	3.7 ± 11.3	4.4 ± 12.9	2.2 ± 6.8	0.596
Excessive force - suture breakage	0.3 ± 1.0	0.4 ± 1.2	0.1 ± 0.2	0.278
Total number of knots	2.1 ± 1.7	2.3 ± 1.9	1.6 ± 0.9	0.246
Suture Score (1–7)	4.8 ± 1.2	4.2 ± 0.9	6.1 ± 0.3	< 0.001

Values are presented as mean ± standard deviation

p values were obtained using the Mann-Whitney test

¹ Scores were automatically generated by the native simulation platform

Time variables are expressed in minutes, except total suturing time, which is expressed in seconds

Groups were defined according to the final Suture Score, the primary outcome of the study

**Table 2:** Correlations between training trajectory variables and final suturing task outcomes

Training trajectory variable	Total suturing time	Accurate needle passages, (%)	Unnecessary needle piercing points	Knot tail length deviation, mm	Time outside visible field	Excessive force - suture breakage	Total number of knots	Suture Score (1–7)
Time in Basic, min	0.020 (0.880)	0.043 (0.753)	0.062 (0.645)	0.061 (0.653)	0.042 (0.754)	-0.084 (0.535)	0.019 (0.889)	-0.010 (0.943)
Repetitions in Basic	-0.053 (0.698)	0.063 (0.641)	-0.003 (0.981)	0.076 (0.572)	0.028 (0.836)	-0.159 (0.239)	0.026 (0.848)	0.032 (0.816)
Mean score in Basic¹	**-0.269 (0.043)**	0.203 (0.129)	-0.139 (0.304)	-0.016 (0.909)	-0.246 (0.065)	-0.171 (0.202)	-0.078 (0.565)	0.277 (0.037)
Time in Essential, min	0.030 (0.823)	-0.069 (0.608)	-0.073 (0.591)	0.026 (0.846)	0.174 (0.196)	0.019 (0.886)	0.050 (0.714)	0.012 (0.929)
Repetitions in Essential	-0.032 (0.814)	-0.056 (0.681)	-0.109 (0.420)	0.036 (0.789)	0.121 (0.371)	-0.075 (0.581)	0.069 (0.608)	0.040 (0.766)
Time in Fundamentals, min	**0.274 (0.039)**	-0.180 (0.180)	0.129 (0.337)	-0.046 (0.735)	0.080 (0.553)	-0.013 (0.921)	-0.155 (0.248)	-0.233 (0.081)
Repetitions in Fundamentals	0.217 (0.105)	-0.173 (0.199)	0.145 (0.283)	-0.059 (0.660)	0.150 (0.265)	-0.070 (0.605)	-0.163 (0.227)	-0.205 (0.125)
Mean score in Fundamentals¹	-0.325 (0.014)	0.118 (0.380)	-0.038 (0.781)	-0.026 (0.847)	0.007 (0.958)	-0.097 (0.472)	-0.042 (0.759)	0.159 (0.238)
Total simulation time, min	0.125 (0.354)	-0.175 (0.192)	0.072 (0.595)	-0.101 (0.454)	0.019 (0.886)	0.018 (0.892)	-0.041 (0.763)	-0.201 (0.134)
Basic time, %	-0.115 (0.394)	0.122 (0.367)	0.009 (0.950)	0.187 (0.165)	-0.054 (0.692)	-0.098 (0.470)	0.026 (0.849)	0.057 (0.672)
Essential time, %	0.029 (0.832)	-0.070 (0.604)	-0.079 (0.560)	0.068 (0.616)	0.190 (0.156)	0.088 (0.515)	0.027 (0.845)	-0.005 (0.973)
Fundamentals time, %	0.224 (0.093)	-0.116 (0.389)	0.080 (0.556)	-0.009 (0.948)	0.130 (0.336)	0.069 (0.611)	-0.195 (0.147)	-0.219 (0.102)

Values are presented as Spearman correlation coefficient r (p value)

¹ Scores were automatically generated by the native simulation platform

Statistically significant correlations (*p* < 0.05) may be highlighted in bold in the final formatted version

### Sample profile and training characteristics

The sample comprised 57 participants. Considering the overall dataset, the mean total simulation time was 642.1 ± 268.7 min. Regarding the distribution of this time across training modules, the mean time spent was 362.9 ± 207.6 min in the Basic module, 118.9 ± 207.7 min in the Essential module, and 109.4 ± 82.1 min in the Fundamentals module, corresponding proportionally to 51.0 ± 17.0%, 13.0 ± 12.5%, and 15.2 ± 9.5% of the total simulation time, respectively. The mean number of repetitions was 110.1 ± 54.7 in Basic, 23.1 ± 19.7 in Essential, and 31.5 ± 26.7 in Fundamentals. The mean scores achieved during the training stages were 83.6 ± 8.0 in Basic and 66.5 ± 19.6 in Fundamentals, as shown in Table 1.

Regarding performance in the final task, the mean total suturing time was 356.6 ± 337.8 s, with a mean percentage of accurate needle passages of 81.8 ± 26.5, a mean number of unnecessary needle piercing points of 8.9 ± 13.0, a mean knot tail length deviation of 28.4 ± 30.6 mm, a mean time with the needle outside the visible field of 3.7 ± 11.3, a mean of 0.3 ± 1.0 episodes of excessive force resulting in suture breakage, a mean total number of knots of 2.1 ± 1.7, and a final Suture Score of 4.8 ± 1.2, as presented in Table 1.

### Comparison between suturing performance groups

After stratification according to the final Suture Score, 39 participants were classified in the group with scores from 1 to 5 (68.4%) and 18 in the group with scores from 6 to 7 (31.6%). Variables related to the prior training trajectory did not show statistically significant differences between groups. There were no differences in time spent in Basic, number of repetitions in Basic, or mean score in Basic (*p* > 0.05). Similarly, no differences were observed for time spent in Essential, number of repetitions in Essential, time spent in Fundamentals, number of repetitions in Fundamentals, mean score in Fundamentals, total simulation time, or the proportion of time spent in Basic, Essential, and Fundamentals (all *p* > 0.05), as described in Table 1.

As expected, participants with higher final scores showed better performance in the components that make up the Suture Score itself. Analysis of these components indicated that group discrimination occurred predominantly in metrics related to temporal efficiency and technical precision, particularly lower total suturing time (*p* < 0.001), a higher percentage of accurate needle passages (*p* < 0.001), and a lower number of unnecessary needle piercing points (*p* = 0.001). In contrast, no statistically significant differences were observed for variables such as time with the needle outside the visible field (*p* = 0.596), excessive force resulting in suture breakage (*p* = 0.278), knot tail length deviation (*p* = 0.319), and total number of knots (*p* = 0.246), suggesting a lower relative contribution of these domains to group discrimination in this sample, as shown in Table 1.

### Association between pedagogical trajectory and final performance

The correlation matrix showed a predominance of weak associations between pedagogical trajectory variables and suturing task outcomes, with most coefficients close to zero and without statistical significance. In particular, basic training measures were not consistently associated with final performance, as observed for time spent in Basic with total suturing time (*r* = 0.020; *p* = 0.880) and with final Suture Score (*r* = -0.010; *p* = 0.943), as well as for number of repetitions in Basic with final Suture Score (*r* = 0.032; *p* = 0.816). Similarly, total simulation time did not show statistically significant associations with the main analyzed outcomes, including total suturing time (*r* = 0.125; *p* = 0.354) and final Suture Score (*r* = -0.201; *p* = 0.134), as demonstrated in Table 2.

As shown in Table 2, statistically significant associations were concentrated in mean performance scores from the early training stages. Mean score in the Basic module was negatively correlated with total suturing time (*r* = -0.269, *p* = 0.043) and positively correlated with the final Suture Score (*r* = 0.277, *p* = 0.037), indicating that better early performance was associated with faster and higher-quality suturing. Mean score in the Fundamentals module was also negatively correlated with total suturing time (*r* = -0.325, *p* = 0.014). Notably, longer time spent in the Fundamentals module was associated with longer total suturing time (*r* = 0.274, *p* = 0.039), reinforcing that additional time-on-task did not, by itself, translate into better performance. In contrast, and as detailed in Table 2, cumulative exposure variables were not significantly associated with final performance, including total simulation time (*r* = 0.125, *p* = 0.354 for suturing time; *r* = -0.201, *p* = 0.134 for Suture Score) and number of repetitions in Basic (*r* = 0.032, *p* = 0.816 for Suture Score).

For the remaining technical components assessed in the suturing task, no statistically significant associations were observed with training time and score variables, including percentage of accurate needle passages, number of unnecessary needle piercing points, knot tail length deviation, time with the needle outside the visible field, excessive force resulting in suture breakage, and total number of knots (all *p* > 0.05), as shown in Table 2.

## Discussion

The main finding of this study was that quantitative exposure variables, such as total simulation time and number of repetitions, were not consistently associated with final suturing performance when interpreted as isolated cumulative measures. In contrast, mean performance scores obtained during earlier training stages showed the most relevant associations. Specifically, better mean performance in the Basic and Fundamentals modules was associated with shorter suturing time, and better performance in the Basic module was also associated with a higher final Suture Score. These findings suggest that, within robotic simulation training, early performance quality may provide more meaningful information than cumulative exposure alone.

These findings can be interpreted through established theories of skill acquisition. The observation that early performance quality predicts final proficiency aligns with Ericsson’s theory of deliberate practice, which holds that expert performance develops not from the mere accumulation of exposure but from structured, goal-directed engagement in which learners actively process feedback and refine performance [16]. This suggests that trainees who process information effectively early in training develop more robust mental models, facilitating the transfer of skills to more complex tasks. Such a quality-centric approach appears more predictive than mere training duration, which may reflect passive repetition rather than active skill acquisition. Cognitive load theory offers a complementary mechanism: by consolidating foundational psychomotor schemas early, high-performing trainees may free working-memory capacity for the higher-order integration demanded by suturing, whereas trainees who rely on repetition without efficient schema formation may remain cognitively overloaded during complex tasks [14, 15]. Under both frameworks, early performance quality — rather than cumulative training time — emerges as the more informative marker of readiness to progress.

However, this association should not be interpreted simply as evidence that training time is unimportant. Rather, it may indicate that trainees differ in their initial ability to adapt to the robotic environment. Participants who achieved higher scores in early simulator tasks may have had greater baseline aptitude for robotic psychomotor skills and faster adaptation to the console interface, or previous technical experience that facilitated early performance. Conversely, participants with lower early scores or longer time spent in foundational stages may represent a subgroup with greater difficulty adapting to the robotic platform. In this sense, early simulator metrics may function as practical markers of individual learning profiles, helping educators identify trainees who may require additional supervised exposure, targeted feedback, and individualized progression before advancing to more complex tasks.

One important contribution of the present study is that it examines real-world retrospective training trajectories in a Brazilian center that did not apply a fully adaptive, performance-based progression algorithm during the study period. This is particularly relevant because, in Brazil, robotic surgery training is interpreted within the national training and credentialing framework proposed by the Brazilian College of Surgeons and the Brazilian Medical Association (CBC/AMB), in which simulation is formally situated within the preclinical phase of training [29, 30]. This phase includes documentation of sequential and progressive psychomotor skills training, suturing practice, operative-skills simulation, and simulated surgical procedures, with records submitted by the trainee, endorsed by the tutor, and homologated by the CBC Commission [29, 30]. In this context, many training environments still rely, at least in part, on exposure, repetition, or documentation of completed training activities as markers of progression. In our sample, neither total simulation time nor repetition counts showed consistent associations with final suturing performance when analyzed as global cumulative variables, reinforcing the view that exposure alone does not adequately capture proficiency. Therefore, the educational value of additional exposure likely depends on whether it is individualized, supervised, and linked to objective performance deficits.

A comparison between the earlier and more recent CBC evaluation forms also shows an important evolution in the way simulation training is documented. The earlier form adopted a more granular and platform-specific structure, listing individual simulator exercises and requiring repeated registration of score and date across basic, advanced, and surgical skills, including camera control, clutching, EndoWrist manipulation, energy control, needle control, needle driving, and multiple suturing tasks [29]. In contrast, the more recent evaluation form adopts a broader institutional documentation model, organized around training phases, general categories of simulation activities, start and completion dates, supporting records, tutor validation, and final homologation [30]. This transition suggests a shift from a checklist centered on predefined simulator tasks toward a more flexible documentation framework that can accommodate different platforms and institutional curricula while preserving the expectation of progressive, supervised preclinical training. Within this context, the present study provides relevant empirical support for refining how this stage is evaluated: simulator-derived performance metrics may help distinguish trainees who are ready to progress from those who require additional exposure and targeted remediation. Therefore, incorporating objective performance indicators into institutional documentation and progression criteria may strengthen the alignment between Brazilian credentialing requirements and proficiency-based robotic surgical education.

Our findings also align with proficiency-based progression frameworks. De Groote et al. and Mazzone et al. previously showed that curricula tied to objective performance standards can produce more efficient and safer learning than traditional or self-directed models [18, 19]. Radi et al. further demonstrated that a mastery-based virtual reality robotic curriculum was feasible, effective, and transferable, with most residents reaching proficiency in basic simulator tasks and showing improvement in inanimate exercises as well [31]. In theoretical terms, this supports the concept described by Satava et al., who defined proficiency as the ability to perform tasks with precision, efficiency, and consistency at or above benchmark levels established by expert validators [32]. In light of this framework, early simulator performance may be understood not only as a measure of current skill, but also as a tool to determine who is ready to progress and who may benefit from additional structured training.

The association between better mean scores in Basic and Fundamentals and shorter suturing time is also conceptually plausible. Basic tasks typically emphasize platform control, depth perception, clutch use, bimanual coordination, and other foundational psychomotor skills, whereas complex tasks such as suturing require integration of force control, angulation, auxiliary arm positioning, needle handling, and technical precision. Sanford et al. demonstrated that early substeps within robotic suturing, such as needle positioning and entry angle, directly influence later substeps and overall task success [23]. Our data extend this logic by suggesting that average performance in earlier simulator stages, even before the final suturing task itself, may already capture relevant aspects of technical readiness. Therefore, lower performance in these early stages may help identify trainees who need reinforcement before being exposed to more technically demanding tasks.

At the same time, not all prior training variables were associated with the final Suture Score, and this deserves careful interpretation. First, the final Suture Score is a native composite metric generated by the simulator platform on the basis of predefined benchmarks. Accordingly, it reflects a weighted combination of different technical dimensions, not a single isolated domain. In our sample, the variables that most clearly differentiated higher- and lower-scoring participants were total suturing time, percentage of accurate needle passages, and unnecessary needle piercing points, whereas time outside the visible field, excessive-force suture breakage, knot tail deviation, and total number of knots did not differ significantly between groups. This pattern suggests that temporal efficiency and needle precision may have had greater discriminative weight than other domains in this specific setting.

Another useful comparison comes from Malpani et al., who found that automated virtual reality teaching cues improved specific technical elements such as needle grasp angle, whereas the control group achieved greater gains in time and motion efficiency [33]. Their findings highlight that technical refinement and temporal efficiency do not always evolve in parallel. This distinction is relevant to the present study because it helps explain why some training variables may show associations with suturing time without necessarily showing equally strong associations with every other technical component of the final task. It also reinforces the importance of analyzing both composite outcomes and their underlying components, particularly when attempting to identify which trainees need additional exposure and which specific skills should be targeted.

Our results also resonate with work on adaptive curricula. Mariani, Pellegrini, and De Momi described a performance-driven adaptive robotic curriculum in which the sequence of tasks was adjusted in real time according to trainee performance, leading to better outcomes than a self-organized training approach [20]. The rationale behind adaptive progression is that learning is not linear, and that learners differ in how quickly they consolidate psychomotor and procedural skills. Although the present study does not test an adaptive curriculum directly, the pattern observed here supports its educational logic: early objective performance appears to contain useful information for progression, particularly because it may help identify trainees who require more supervised exposure, targeted remediation, and delayed progression before advancing to complex tasks.

An additional strength of the present study is its ecological relevance. Much of the literature in robotic simulation is based on intervention trials, validation studies, or tightly structured curricula, and recent systematic reviews have highlighted the persistent heterogeneity of training models and the limited validation of assessment tools across specialties [34]. By contrast, the present work examines how trainees actually behaved in a real training center under routine institutional conditions. This helps address an important gap in the literature, namely the limited availability of studies evaluating how learners build their pedagogical trajectory when training is not strictly dictated by a predefined adaptive curriculum. In that sense, the present study helps bridge the gap between idealized curricular design and the actual behavior of trainees in routine educational settings. It also suggests that routinely collected simulator data may be useful not only for retrospective evaluation, but also for the early recognition of trainees who need a more individualized training pathway.

Some limitations of this study should be acknowledged. First, its retrospective design precludes causal inference, allowing only the identification of associations rather than cause-and-effect relationships. Second, the study was conducted in a single training center, which may restrict the generalizability of the findings to other institutions and curricular structures. Third, all data were derived from a single simulator platform; because performance metrics and their calibration are platform-defined, the results may not be directly reproducible across different hardware or software environments. Fourth, the sample comprised 57 participants, a size that, while adequate for the primary analyses, may have limited the statistical power to detect smaller effects and weaker associations. Fifth, detailed demographic and background data — including age, sex, surgical specialty, training level, and previous laparoscopic, robotic, or simulation experience — were not consistently available and therefore could not be controlled for. Sixth, the study lacked external validation, as simulator-derived performance was not correlated with real-world clinical or intraoperative outcomes; consequently, although suturing tasks are commonly used as proxies for advanced robotic technical skill, transferability to dry-lab exercises, supervised clinical training, or operative performance cannot be assumed directly [6, 7, 22]. Seventh, only participants who completed the training trajectory were included, which may have introduced a survival bias by excluding trainees who discontinued training. Eighth, the analysis was essentially cross-sectional with respect to the training trajectory available in the institutional database, without longitudinal tracking of individual learning curves or assessment of skill retention over time. Finally, unmeasured confounding cannot be excluded, as factors such as motivation, fatigue, and innate psychomotor aptitude were not captured; this is particularly relevant because early simulator performance may partly reflect baseline individual characteristics, including previous technical experience or natural ability to adapt to the robotic console. In addition, the pedagogical trajectory analysis was restricted to the native Basic, Essential, and Fundamentals modules, while more complex tasks were excluded to reduce specialty-related bias; although this improved comparability across participants, it also limited the analysis to part of the overall robotic training experience.

Despite these limitations, the study offers a meaningful contribution to robotic surgical education. Rather than testing whether one predefined curriculum outperforms another, it evaluates whether naturally occurring differences in pedagogical trajectory are associated with advanced simulator performance. The overall pattern suggests that early performance quality, particularly average performance in initial stages, may help identify trainees with greater difficulty adapting to the robotic environment. From a pedagogical perspective, this supports a shift from interpreting simulation time as a simple cumulative requirement toward using early objective metrics to guide individualized training. Trainees with lower early performance may benefit from additional supervised exposure, targeted feedback, and delayed progression before advancing to more complex tasks. Future studies should investigate whether these findings can be replicated in multicenter datasets and whether specific early metrics can serve as stronger predictors of later proficiency. Importantly, future research should also collect detailed baseline characteristics of trainees, including previous laparoscopic experience, prior exposure to robotic platforms, simulation experience, surgical specialty, and training level. Such data may help clarify whether early simulator performance is primarily associated with previous technical experience or whether, in the absence of such associations, it may reflect individual aptitude or natural adaptability to the robotic environment. This distinction may be important for designing adaptive progression models that not only measure performance, but also explain why some trainees require different levels of exposure, feedback, and remediation before progressing to complex tasks.

## Conclusion

In this study, cumulative exposure metrics, including total simulation time and number of repetitions, were not consistently associated with performance in a complex robotic suturing task when analyzed as isolated measures. In contrast, mean performance scores obtained during early simulator stages were more closely related to subsequent suturing outcomes, with better early performance in the Basic and Fundamentals modules associated with shorter suturing time and higher final Suture Scores. These findings suggest that basic simulation tasks may be understood not only as preparatory exercises for more complex robotic skills, but also as early markers of adaptability to robotic surgery training. From a pedagogical perspective, early performance in foundational tasks may help identify trainees who adapt more readily to the robotic environment, as well as those who experience greater difficulty and may require additional supervised exposure, targeted feedback, and individualized progression before advancing to complex modules. Therefore, simulator-derived metrics from basic tasks may support a shift from uniform, time-based training pathways toward more adaptive and performance-guided educational trajectories.

These findings should be interpreted in light of the study’s limitations, particularly its retrospective design and single-center sample, which warrant confirmation in larger, prospective cohorts. Future prospective and multicenter studies should evaluate whether the early identification of these learning profiles, followed by tailored remediation strategies, improves skill acquisition and retention, and whether these simulation-derived metrics correlate with actual clinical outcomes in the operating room.

## Supplementary Information

Supplementary Material 1

## Data Availability

No datasets were generated or analysed during the current study.
