# Supplementary material for: Quality over quantity: early performance predicts suturing proficiency in robotic surgery simulation
Source: J Robot Surg. 2026 Jul 21;20(1):735. doi: 10.1007/s11701-026-03702-0 (PMC13385081; doi:10.1007/s11701-026-03702-0)
Supplement: Supplementary file 1 — Supplementary Material 1 [file 11701_2026_3702_MOESM1_ESM.docx]

**Supplementary Table S1.** Data dictionary of simulator-derived variables

| **Variable** | **Definition** | **Unit** | **Type** | **Analytical role** |
| --- | --- | --- | --- | --- |
| **User ID** | Unique identifier assigned to each participant | — | Categorical |  |
| **Total simulation time** | Total cumulative time spent across all simulator modules (Basic, Essential, Fundamentals) | Minutes | Continuous | Training exposure |
| **Time in Basic** | Total time spent in tasks belonging to the native Basic module | Minutes | Continuous | Training exposure |
| **Repetitions in Basic** | Number of completed task repetitions in the Basic module | Count | Discrete | Training exposure |
| **Mean score in Basic** | Mean simulator-generated performance score across Basic module tasks | Platform score | Continuous | Training performance |
| **Time in Essential** | Total time spent in tasks belonging to the Essential module | Minutes | Continuous | Training exposure |
| **Repetitions in Essential** | Number of completed task repetitions in the Essential module | Count | Discrete | Training exposure |
| **Time in Fundamentals** | Total time spent in tasks belonging to the Fundamentals module | Minutes | Continuous | Training exposure |
| **Repetitions in Fundamentals** | Number of completed task repetitions in the Fundamentals module | Count | Discrete | Training exposure |
| **Mean score in Fundamentals** | Mean simulator-generated performance score across Fundamentals module tasks | Platform score | Continuous | Training performance |
| **Basic time (%)** | Percentage of total simulation time allocated to the Basic module | % | Continuous | Training distribution |
| **Essential time (%)** | Percentage of total simulation time allocated to the Essential module | % | Continuous | Training distribution |
| **Fundamentals time (%)** | Percentage of total simulation time allocated to the Fundamentals module | % | Continuous | Training distribution |
| **Total suturing time** | Total time required to complete the final suturing task | Seconds | Continuous | Secondary outcome¹ |
| **Accurate needle passages** | Percentage of needle passes performed through predefined optimal targets | % | Continuous | Secondary outcome¹ |
| **Unnecessary needle piercing points** | Number of unintended or non-optimal tissue punctures during suturing | Count | Discrete | Secondary outcome¹ |
| **Knot tail length deviation** | Deviation of knot tail length from predefined optimal value | Millimeters | Continuous | Secondary outcome¹ |
| **Time outside the visible field** | Total time the needle or instruments remain outside the endoscopic visual field | Seconds | Continuous | Secondary outcome¹ |
| **Excessive force – suture breakage** | Number of events where excessive force caused suture breakage | Count | Discrete | Secondary outcome¹ |
| **Total number of knots** | Total number of knots executed during the suturing task | Count | Discrete | Secondary outcome¹ |
| **Suture Score (1–7)** | Composite simulator-generated score (including time, accuracy, unnecessary punctures, knot quality, visibility control, force, and knot count) | Score (1–7) | Ordinal | **Primary outcome** |

¹ Component of Suture Score
